# Antiviral Activity of Angelica Tenuissima Nakai against Influenza A Virus

**DOI:** 10.3390/pathogens13090761

**Published:** 2024-09-04

**Authors:** Won-Jong Park, Gi-Sang Bae, Youn-Ho Han

**Affiliations:** 1 Department of Oral and Maxillofacial Surgery, Seoul St. Mary’s Hospital, College of Medicine, The Catholic University of Korea, Seoul 06591, Republic of Korea; roll8888@naver.com; 2 Department of Pharmacology, School of Korean Medicine, Wonkwang University, Iksan 54538, Republic of Korea; baegs888@wku.ac.kr; 3 Department of Oral Pharmacology, College of Dentistry, Wonkwang University, Iksan 54538, Republic of Korea

**Keywords:** Angelica tenuissima Nakai, antiviral, influenza A virus

## Abstract

The influenza A virus poses a serious threat to human health and is an important global public health issue. The drugs currently used for treatment are becoming increasingly ineffective against influenza A viruses and require the development of new antiviral drugs. Angelica tenuissima Nakai (ATN), a traditional herbal medicine belonging to the Umbelliferae family, exhibits a broad range of pharmacological activities, including inflammation, headache, and cold symptoms. In the present study, based on target protein identification, functional enrichment analysis, and gene set comparisons, we first suggested that ATN has potential therapeutic effects against influenza A virus infection. Next, methylthiazol tetrazolium (MTT) and sulforhodamine B colorimetric (SRB) assay results revealed that ATN exhibited low cytotoxicity in Madin–Darby canine kidney (MDCK) cells. The antiviral properties of ATN were observed against H1N1 and H3N2 virus strains. Microscopy confirmed the increased survival rate of the host cells. Further time-of-addition experiments revealed that the addition of ATN before virus adsorption showed similar results to the whole period of treatment. The pre- and co-treated groups showed lower levels of viral RNA (M1 protein). The results of this study suggest that ATN exhibits antiviral properties against the influenza A virus. These therapeutic properties of ATN can serve as a theoretical basis for further research on the applicability of ATN in the development of antiviral agents.

## 1. Introduction

Viral diseases range from simple treatable infections to infectious diseases, that often turn the world upside down. Therefore, it is unreasonable to control the epidemiological and pathological changes of the virus in a simple manner, as each virus has its own characteristics and represents a different problem [1]. Typically, influenza A viruses are highly contagious and a major cause of recurrent infectious diseases and pandemics. Influenza A viruses possess a segmented negative-sense RNA genome that encodes viral proteins. Hemagglutinin (HA) and neuraminidase (NA) are the best-characterized viral proteins that are used to categorize influenza A viruses into different strains [2]. Influenza A viruses have been evolving and circulating among their animal and human hosts since the pandemic influenza outbreak in 1918, causing substantial mortality and morbidity [3].

Currently, vaccines and antiviral drugs are used for the prevention and treatment of influenza [4,5]. Vaccines are used as basic means to prevent viral infections; however, they require periodic monitoring and have the disadvantage of only approximately 50% effective in elderly patients with high mortality [6]. Currently, three types of antiviral agents are available for influenza treatment: M2 channel inhibitors, neuraminidase inhibitors, and the recently approved endonuclease inhibitor including baloxavir marboxil [7,8]. However, the use of these drugs is limited by the emergence of drug-resistant viruses [9,10]. Furthermore, to reduce human health risks in the current rapidly changing environment, economic and groundbreaking strategies and responses related to viral diseases are required. The development of safe and effective antiviral drugs is a top priority in drug development, and many studies have focused on developing naturally derived anti-influenza agents and traditional herbal medicines [11].

Angelica tenuissima Nakai (ATN), belonging to the family Apiaceae/Umbelliferae, is a traditional medicinal plant in East Asia that is used for various treatments, including headache, toothache, and diarrhea. The methanol extract of ATN exhibits antioxidant, liver protective, and anti-inflammatory activities, as indicated by the inhibition of cyclooxygenase-2 [12,13]. Although ATN has been used to alleviate flu-like symptoms, no specific mechanism against influenza A virus has been identified. This study investigated the antiviral effect of ATN against influenza A virus to understand the applicability of ATN for the management of influenza infection.

## 2. Materials and Methods

### 2.1. Target Protein Construction of ATN

To construct a target protein of ATN, genes with co-occurrence for ATN were gathered through PubChem (https://pubchem.ncbi.nlm.nih.gov/, accessed on 12 May 2024). In total, 495 genes associated with the ATN removing duplicates were sorted through STRING database (http://www.string-db.org/, accessed on 12 May 2024) with a score ≥ 0.7, which represents high confidence.

### 2.2. Enrichment Analysis

Functional Enrichment Analysis was conducted to analyze the target protein construction of ATN, using Cytoscape String App, based on virus perturbation analysis. Virus perturbations from Gene Expression Omnibus (GEO) refers to the databases of how external factors or interventions affect the behavior and function of viruses, which involves experimental or computational approaches aimed at manipulating viral components, conditions, or host-virus interactions to understand the consequences and mechanisms of viral perturbation. The analysis was performed to predict potential pathways of ATN.

### 2.3. Gene Set Construction

Using GeneCards (http://www.genecards.org/, accessed on 15 May 2024) [14]. and DisGeNet (http://www.disgenet.org/, accessed on 15 May 2024) [15]. influenza A virus-related genes were collected to create gene sets. 3280 genes were gathered from GeneCards and 563 genes were gathered from DisGeNet. Based on the genes from each database, three different influenza A virus gene sets: GeneCards, DisGeNet, and sum of those. The summed gene set contains 3461 genes, which indicates 211 genes were in common in two different.

### 2.4. Preparation of Angelica Tenuissima Nakai Roots Extract

ATN roots was purchased from Kwang Myung Dang (Ulsan, Republic of Korea) and verified at the Department of Herbology, College of Korean Medicine, Wonkwang University. Extraction was performed using the following method: after adding 1000 mL distilled water to 75 g of ATN, the mixture was subjected to decoction formation for 2 h at 100 °C, followed by enrichment and freeze-drying process to obtain the ATN extract. The yield of dried extract was 13.7 g. Several key components of ATN including ferulic acid, ligustilide, caffeic acid, eleutheroside B, and oregano acid, have been identified through HPLC [16].

### 2.5. Propagation of Influenza A Virus in Madin–Darby Canine Kidney (MDCK) Cell Line

Influenza A/H1N1/2009/CA was provided by the Korean Centers for Disease Control and Prevention. Influenza A/H3N2, isolated from humans in 2014, was donated by the Korean National Research Resource Center (Seoul, Republic of Korea; registration number: KBPV-VR-85). MDCK cells were grown in Dulbecco’s modified Eagle’s medium (DMEM; Gibco BRL, Grand Island, NY, USA) supplemented with 2 mM of L-glutamine, 0.1 mM non-essential amino acid mixture, 100 U/mL penicillin, 100 μg/mL streptomycin (Thermo Fisher Scientific, Waltham, MA, USA), and 10% fetal bovine serum (Gibco BRL). All cells were cultured as monolayers and incubated at 37 °C in 5% CO_2_.

### 2.6. Evaluation of In Vitro Cytotoxicity of ATN

The cytotoxicity of ATN against MDCK cells was determined using the methylthiazol tetrazolium (MTT) and SRB (Sulforhodamine B colorimetric) assay, which was performed for 48 h using various concentrations of ATN. The 50% cytotoxic concentration (CC50) values were calculated using Prism 5.03 software (GraphPad Software, San Diego, CA, USA).

### 2.7. Hemagglutination Assay

Influenza A viruses (H1N1 and H3N2) cultured in MDCK cells were used to evaluate the effectiveness of ATN. To compare the titers of the influenza A virus, a hemagglutination assay was used. A round-bottomed 96-well dish was prepared as follows. In the first column of the plate, 50 µL of virus sample was added, and wells in the subsequent columns were filled with 2-fold serially diluted samples. After adding serial dilutions of the virus samples, chicken red blood cells (0.5%, 50 µL) were added to each well and mixed gently. The plates were incubated for 30 min at room temperature. Following the incubation period, an assay was performed to distinguish between agglutinated and non-agglutinated wells.

### 2.8. Determination of Influenza A Virus Median Tissue Culture Infectious Dose (TCID50)

Influenza virus was 10-fold diluted and added to a 96-well plate containing 2 × 10^4^ MDCK cells/well. Infected cells were incubated at 37 °C for 72 h. The endpoint was determined when the cytopathic effect appeared. The titer was calculated using the Reed–Muench method.

### 2.9. Hemagglutination Inhibition (HI) Assay

The attachment between the virus and ATN was evaluated using a HI assay. Influenza A virus H1N1 or H3N2 [multiplicity of infection (MOI) = 0.5] was pre-incubated with ATN (0.8 to 100 μg/mL) at 4 °C for 1 h. The titer of ATN-treated virus was determined using a hemagglutination assay.

### 2.10. Microscopic Examination of Virus-Infected Cells

MDCK cells in 24-well plates were pre-treated with various concentrations of ATN (50–800 μg/mL) for 2 h and then infected with influenza A viruses H1N1 and H3N2 (MOI = 0.5). Microscopic examination was performed 24 hpi using a light microscope with a 4× objective lens.

### 2.11. Time-of-Addition Assay

MDCK cells in 6-well plates were infected with influenza A virus H1N1 (MOI = 0.5) between −1 and 0 h for 1 h. The cells were then treated with ATN (100 μg/mL) at five different intervals: −3 to −1 h (pre-treatment), −3 to 9 h (whole period treatment), 0 to 9 h, 3 to 9 h, and 6 to 9 h post-infection (hpi). Supernatants were harvested at 9 hpi. The viral titer was determined using a TCID50 (median tissue culture infectious dose) assay protocol.

### 2.12. Viral RNA Isolation and Real-Time PCR

MDCK cells were infected with influenza A viruses H1N1 and H3N2 (MOI = 0.1) for 1 h and treated with ATN (1 mg/µL) for the indicated period. The cells were harvested at 9 hpi. Real-time PCR was performed as described previously [17]. The primer sequences for M1 were 5′-GAC CAA TCC TGT CAC CTC-3′ (forward) and 5′-GAT CTC CGT TCC CAT TAA GAG-3′ (reverse). The GAPDH primers used were 5′-AAG AAG GTG GTG AAG CAG GC-3′ (forward) and 5′-TCC ACC ACC CTG TTG CTG TA-3′ (reverse).

### 2.13. Statistical Analysis

Statistical analysis was performed using Prism 5.03 software (GraphPad Software). One-way ANOVA with Tukey’s post-hoc test was used. Statistical significance was set at *p* < 0.05.

## 3. Results

### 3.1. Target Proteins of ATN and Functional Enrichment Analyses of Targets

**Figure 1:**
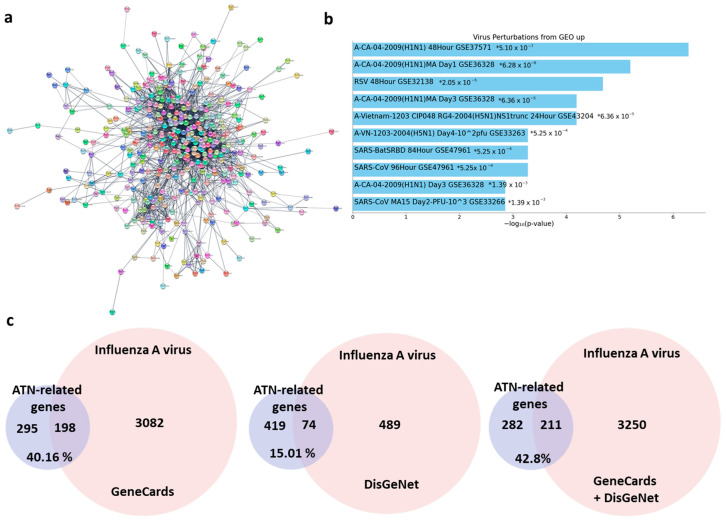
ATN-related gene network and the comparison between the ATN-related gene and the Gene Sets of influenza A virus. (**a**) Network diagram of ATN-related genes using Cytoscape visualization software. (**b**) Top 10 pathways with the lowest *p*-value in virus perturbations of ATN-related gene. * indicates *p*-value. (**c**) Respective comparison between ATN-related gene, GeneCards Gene Set and DisGeNet Gene Set of influenza A virus.

To clearly confirm the effects of ATN on viral infection from a perspective of target-gene-diseases relationship functional enrichment analyses was performed. In PubChem databases, 821 targets of ATN including peroxiredoxin 5, pyruvate dehydrogenase kinase 1, and amylase α 2a etc. and 495 associated genes were identified, and the network diagram of ATN-related genes was constructed using Cytoscape visualization software version 3.9.1 (Figure 1a). With the Functional Enrichment Analysis, a ATN was expected to affect ‘H1N1’, a type of influenza A virus (GSE37571) at most high rate through virus perturbations from GEO (Figure 1b). To investigate the correlation between ATN and influenza A virus, the gene sets were created based on GeneCards, DisGeNet and sum of those. As shown in Figure 1c, three gene sets of influenza A virus contained 3280, 563, 3461 genes respectively, which are the genes from GeneCards, DisGeNet and a sum of those. Among 76 genes, the ATN shared 198 and 74 common genes with those based on GeneCards and DisGeNet respectively). This represents that 40.16% and 15.01% of ATN genes were related influenza A virus with the summed gene set, ATN had 211 genes in common, implying 42.8% ATN genes have correlation with influenza A virus. These results suggest the possibility as a treatment of ATN on influenza A virus infection based on the focus of protein targets and genes.

### 3.2. ATN Exhibits a Low Cytotoxic Effect on MDCK Cells 

**Figure 2:**
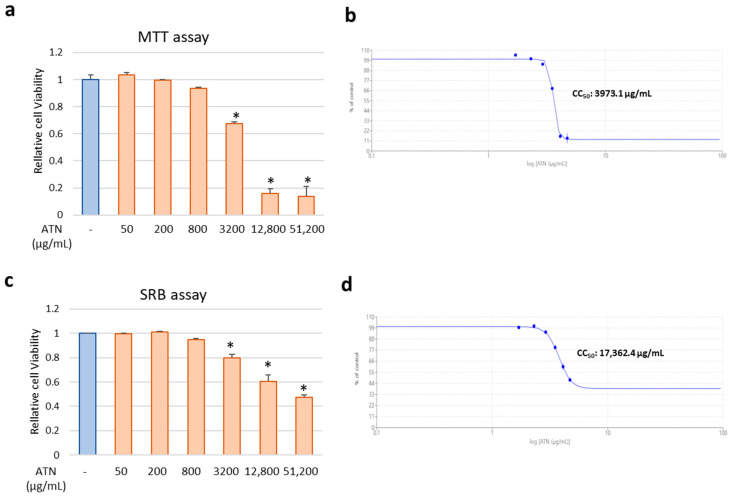
Cytotoxicity of ATN in MDCK cells. MDCK cells were treated with serially diluted ATN (concentrations shown in the figure) and incubated for 48 h. (**a**,**b**) Cell viability was determined using the MTT assay. (**c**,**d**) Cell viability was determined using the SRB assay. The 50% cytotoxic concentration (CC50) values were calculated using Prism 5.03 software. * indicates *p* < 0.05 compared to the ATN non-treated control group (*n* = 3).

The viability of MDCK cells was investigated using the MTT assay to determine the non-toxic concentrations of ATN. Cells were incubated in the presence of 4-fold serial dilutions of ATN for 48 h, and the CC50 was evaluated. The results of the MTT assay confirmed that the cell survival rate decreased by 22% at 3.2 mg/mL of ATN, compared to the control group, and the toxicity was evident at concentrations above 12.8 mg/mL of ATN, whereas the concentration (below 800 μg/mL) used for later efficacy evaluation showed no effect on cell survival (Figure 2a). The cytotoxicity concentration at 50% value (CC50) of ATN was revealed to be 3973.1 μg/mL (Figure 2b). Next, SRB assay was performed to directly measure changes in cell density or mass, providing a complementary assessment of cytotoxicity that is independent of metabolic activity. In the SRB assay, similar to the MTT assay, a slight cytotoxicity was observed at concentrations of 3.2 mg/mL or higher, but the CC50 value found to be remarkably high at 17,362.4 μg/mL, indicating low overall cytotoxicity (Figure 2c,d).

### 3.3. ATN Exhibits Antiviral Property against H1N1 and H3N2 Influenza A Viruses

**Figure 3:**
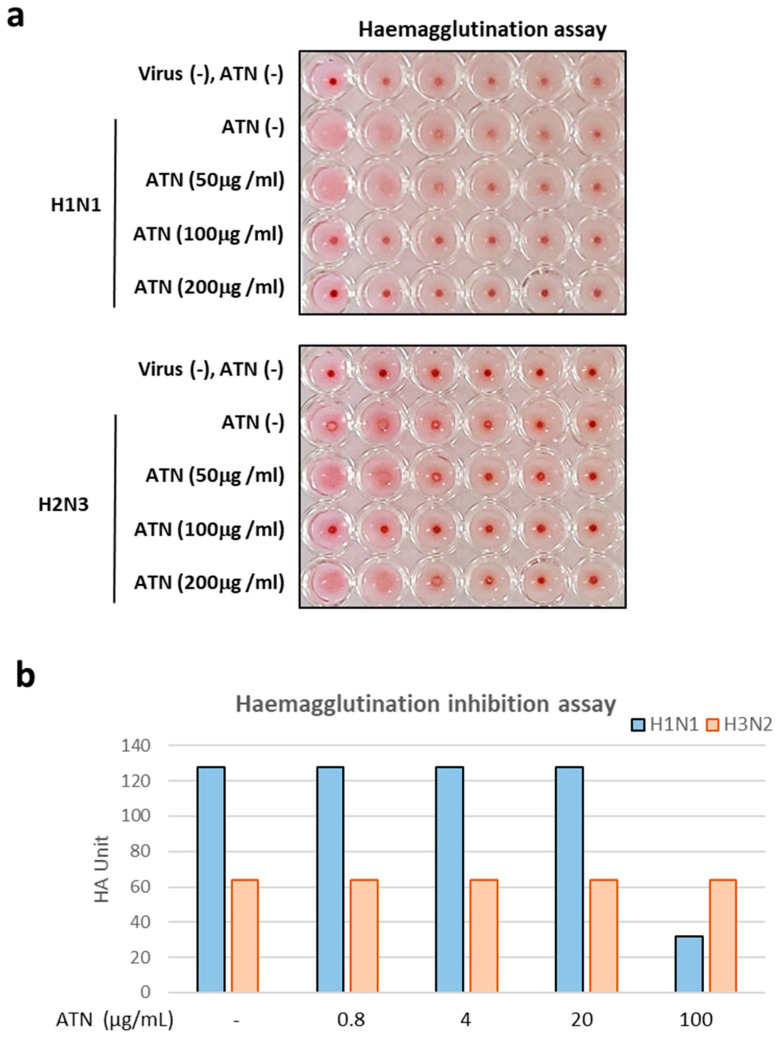
ATN exhibits antiviral property against influenza A viruses of subtypes H1N1 and H3N2. (**a**) ATN showed inhibitory properties at concentrations of 100 and 200 μg/mL against the H1N1 virus and at concentrations of 100 μg/mL against the H3N2 virus. (**b**) Hemagglutination inhibition assay results reveal that H1N1 is directly bound with ATN.

Next, to evaluate the anti-influenza properties of ATN, three different methods were used. The first method involved pre-treatment of MDCK cells with ATN for an hour and washes with phosphate-buffered saline (PBS), and then incubation of the MDCK cells with virus. The second method was to mix ATN and viruses and then incubate the mixture with MDCK cells. Third method used was as follows: after 1 h of infection, the supernatant was washed with PBS and treated with ATN. The concentrations of ATN were 50, 100, and 200 μg/mL, and the MOI was 0.1. All supernatants were collected 24 hpi, and the viral titer was determined using a hemagglutination assay. In the second and third methods, there were no differences from the control group; however, in the first method, ATN exhibited antiviral properties. Inhibition of virus virulence was observed at ATN concentrations of 100, 200 μg/mL for H1N1 and 100 μg/mL for H3N2 (Figure 3a). HI assay (Figure 3b) results revealed the HA unit of H1N1 was reduced at 100 μg/mL, indicating the possibility that ATN binds to the hemagglutinin (HA) of H1N1 directly

### 3.4. ATN Enhances the Cell Survival Rate against H1N1 and H3N2 Influenza A Viruses

**Figure 4:**
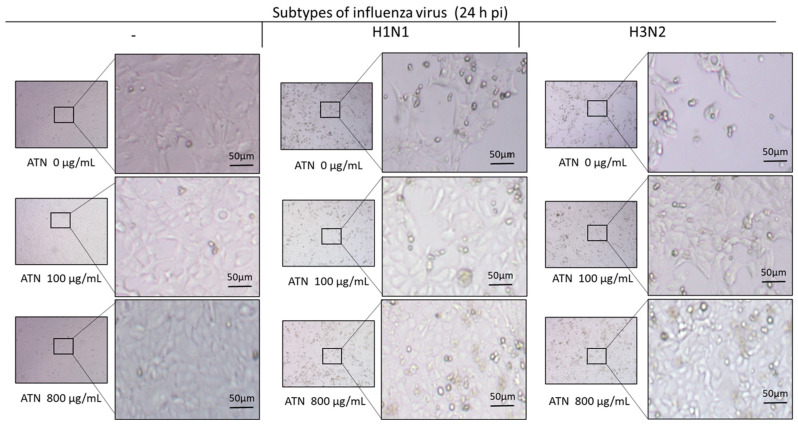
ATN increases the survival rate of cells infected with influenza (H1N1, H3N2) viruses. MDCK cells infected with influenza A virus (MOI = 0.5) were treated with ATN (100 and 800 μg/mL). A greater number of cells survived when the virus was treated with ATN. Microscopic examination was performed 24 h post-infection (hpi) using a microscope with a 4× objective lens (scale bar = 50 μm).

Next, microscopic analysis was conducted to determine whether the survival rate of MDCK cells after influenza infection was recovered by ATN. In the case of non-infected cells, cell survival remained constant, regardless of ATN treatment. In contrast, cells infected with H1N1 or H3N2 showed increased cytotoxicity and debris, which increased cell survival following ATN treatment (100 or 800 μg/mL, respectively) in a dose-dependent manner (Figure 4).

### 3.5. ATN Exhibits Antiviral Activity at an Early Stage of Infection

**Figure 5:**
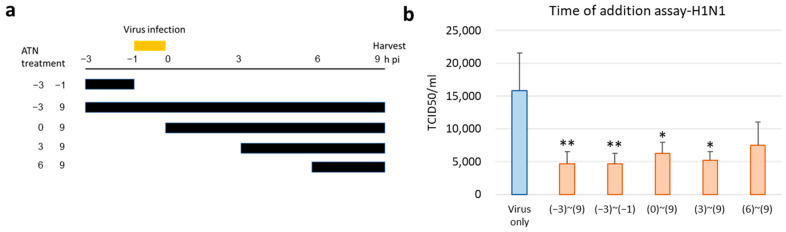
ATN exhibits antiviral activity at an early stage in a time-of-addition assay. (**a**) Schematic diagram of ATN treatment. MDCK cells were infected with H1N1 (MOI = 0.5) at −1 to 0 h. ATN (100 μg/mL) was incubated with the MDCK cells for the durations indicated. All supernatants were collected at 9 h post-infection (hpi) to determine the titers using median tissue culture infectious dose (TCID50). (**b**) The results show that ATN exhibits an antiviral property. * indicates *p* < 0.05 compared to the virus-only group (*n* = 3); ** indicates *p* < 0.01 compared to the virus-only group (*n* = 3).

To verify the antiviral activity of ATN, a time-of-addition assay was performed. Influenza A virus has a life cycle with stages of adsorption, uncoating, biosynthesis, and release of the progenitor virus, and vaccination with the influenza A virus is followed by the above steps after 8–10 h [18]. Therefore, we selected five intervals (−3 to −1, −3 to 9, 0 to 9, 3 to 9, and 6 to 9) for the inhibition time course of ATN treatment (Figure 5a), and H1N1 was used to infect MDCK cells at an MOI of 0.5. As shown in Figure 5b, when ATN was added at the early adsorption stage (−3 to −1), the TCID50 value was notably reduced, similar to the whole period of treatment (−3 to 9), whereas less inhibitory activity was observed when ATN was treated during the other interval stages. 

### 3.6. ATN Decreases Viral RNA Synthesis at Early Stages of Infection

**Figure 6:**
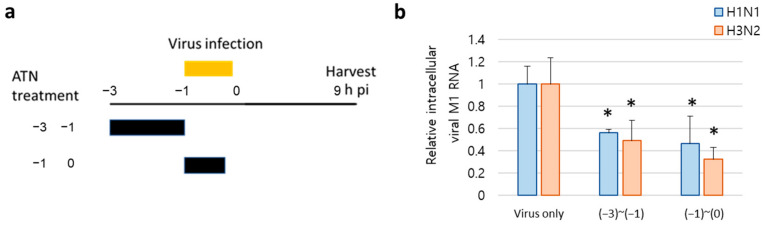
ATN inhibits viral RNA synthesis at an early stage of infection. (**a**) Schematic diagram of ATN treatment. MDCK cells were infected with H1N1 and H3N2 (MOI = 0.1) and treated with ATN (1 mg/mL). All supernatants were collected at 9 h post-infection (hpi). (**b**) Viral RNA expression was detected by real-time PCR using specific primers for M1 protein. * indicates *p* < 0.05 compared to the virus-only group (*n* = 3).

The effect of ATN in the early stages of viral infection was further confirmed by elucidating the intracellular viral mRNA level of the M1 protein. Compared with the virus-only control, ATN (1 mg/mL), added at −3 to −1 (pre-treatment), and −1 to 0 (co-treatment) intervals (Figure 6a), inhibited the mRNA level of the M1 protein gene in both H1N1- and H3N2-infected cells, respectively (Figure 6b). These results indicate that ATN exhibits antiviral activity at an early stage of infection rather than at later stages, including the replication and release processes of the viral lifecycle.

## 4. Discussion

Many modern drugs have been developed to verify the effects of traditional natural products. Verification of the therapeutic effects of a large number of natural substances that are widely used in Asia as traditional remedies could be an important starting point for the design of new drugs [19]. While traditional medicinal substances might have a higher chance of being clinically safer than novel phytochemicals, the identification of their definitive active ingredients remains challenging, as their chemical composition may differ based on geographical location and the source of origin [20]. Among these natural products, ATN is traditionally used to treat several types of pain, including headache, abdominal pain, and toothache, and to treat fever, sputum, and cough due to a cold [13]. Recent studies have identified the active components and biological functions of ATN. For instance, the essential oils of ATN containing 3-butylidenephthalide and 3-butylidene-4,5-dihydrophthalide have the effect of alleviating pain and providing relief from female diseases [21]. In addition, ligustilide and n-butylidenephthalide, isolated from ATN, decreased fatty acid uptake and esterification in mice and showed potential as therapeutics for nonalcoholic fatty liver disease [22]. Meanwhile, ATN was reported to exhibit antiviral effects by increasing interferon production, but its efficacy against influenza A viruses has not yet been identified.

M2 channel inhibitors and neuraminidase inhibitors are approved antiviral drugs available at present [7]. The recent emergence of viruses resistant to these drugs has raised concerns regarding new, potentially pandemic influenza strains [10,23]. Therefore, it is necessary to develop novel antiviral agents that do not cause resistance. The antiviral activity of ATN, which is known to have anti-flu properties, was evaluated in this study. ATN was shown to have an inhibitory effect on H1N1 and H3N2 (Figure 3a). The results of the time-of-addition assay indicated that ATN inhibited the early stages of infection by influenza A virus (H1N1; Figure 5b). ATN is hypothesized to interfere with virus binding, because pre-treatment with ATN has an adequate antiviral effect. However, ATN alone did not appear to bind to RBCs. Therefore, ATN does not attach to the HA receptor in the host cell. Instead, it is thought to cause a change in the binding site of the cell surface. HA is cleaved into HA1 and HA2. HA1 is responsible for cell–virus binding, whereas HA2 catalyzes fusion/genome uncoating via membrane fusion at an early stage of replication [24]. The HI assay revealed that ATN could reduce influenza A virus (H1N1)-induced RBC agglutination (Figure 3b), suggesting that ATN may bind to the HA receptor-binding region (HA1).

Our findings show that ATN reduces viral RNA levels; however, this does not conclusively demonstrate that ATN inhibits viral RNA synthesis specifically. The reduction could also result from ATN affecting other stages of the viral lifecycle, such as viral adsorption or entry into the host cell. To address this, we have revised the manuscript to clarify that while our results indicate a decrease in viral RNA, further experiments are needed to determine whether ATN acts at the RNA synthesis stage or impacts earlier steps of viral replication.

Antiviral drugs that target genetically consistent HA2 proteins (i.e., the stem region of HA proteins) of influenza A viruses have been widely studied [25,26,27]. Antiviral drugs inhibiting virus attachment have also been developed; for example, drugs that interact with sialic acid present on the respiratory epithelial cell surface inhibit the binding of the virus to the host cell surface receptors [28]. Similarly, ATN may suppress viruses by inhibiting cell–virus interactions. As of now, it is difficult to elucidate the active components of ATN that are involved in this mechanism. Further studies are required to identify and characterize the ATN components to aid in the development of new ATN-based antiviral drugs.

## 5. Conclusions

ATN is a traditional medicine used to treat toothaches and flu-like symptoms. However, no studies have been conducted that elucidate the therapeutic effects of ATN. The results of the current study confirmed that ATN exhibits antiviral properties against the influenza virus in the early stages of viral infection. These results indicated the potential of ATN as a treatment regimen for influenza infections.

## Data Availability

Please contact the corresponding author for reasonable data requests.
